# Fine-Tuning Reception in the Bone: PPAR*γ* and Company

**DOI:** 10.1155/PPAR/2006/52950

**Published:** 2006-08-01

**Authors:** Z. Elizabeth Floyd, Sanjin Zvonic, Mark E. Nuttall, Jeffrey M. Gimble

**Affiliations:** ^1^Stem Cell Laboratory, Pennington Biomedical Research Center, Louisiana State University System, Baton Rouge, LA 70808, USA; ^2^Centocor, Johnson and Johnson, Horsham, PA 19044, USA

## Abstract

PPAR*γ* plays a central role in the formation of fat. Regulation of PPAR*γ* activity depends on numerous factors ranging from dietary ligands to nuclear hormone coactivators and corepressors to oxygen-sensing mechanisms. In addition, the interplay of PPAR*γ* with other nuclear hormone receptors has implications for the balance between adipogenesis and osteogenesis in mesenchymal stem cells of the bone marrow stroma. This review will explore a range of factors influencing PPAR*γ* activity and how these interactions may affect osteogenesis.

## INTRODUCTION

This special issue focuses on the latest findings relating to the role of PPARs in bone metabolism. This review uses the broader scope of the nuclear hormone receptor superfamily to assess the relationship between adipogenesis and osteogenesis, both in vitro and in vivo, and their underlying regulatory mechanisms. While PPAR*γ* takes center stage, the vitamin D_3_, estrogen, LXR (liver X receptor), and related receptors are used as examples to explore the potential impact of coactivators and corepressors on bone marrow-derived mesenchymal stem cell (MSC) differentiation. The role of dietary and endogenous ligands, such as genistein, long chain fatty acids, and resveratrol, are evaluated in the context of nuclear receptor regulation of bone physiology and pathology.

Bone marrow stroma MSCs give rise to a number of cell types, including osteoblasts and adipocytes [1, 2]. Bone formation is regulated by Runx2/Cbfa 1, a member of the runt homology domain transcription factor family [3–6] while fat formation depends on the peroxisome proliferator-activated receptor gamma (PPAR*γ*) [7–9]. A number of studies suggest that bone formation is related inversely to adipocyte formation in the marrow cavity [2, 10]. In vitro studies using bone marrow-derived MSCs find that induction of adipocyte differentiation inhibits osteoblastic bone formation [2, 10]. Likewise, agents inducing osteoblast differentiation inhibit adipogenesis [11]. These findings are consistent with the results of Akune et al [12] demonstrating that haploinsufficiency of PPAR*γ* promotes bone formation.

The reciprocal relationship between PPAR*γ* levels and osteogenesis is particularly evident with increased age [12, 13], supporting a role for PPAR*γ* in bone development and osteoporosis associated with aging. The increasing age of the population and osteoporosis associated with aging indicates a need to further explore the regulation of PPAR*γ* with respect to bone formation. The interplay of PPAR*γ* with other nuclear receptors and the regulation of PPAR*γ* by a range of cofactors in other tissue types may offer insights into potential therapeutic targets for regulating bone formation.

## PPAR*γ*: CROSSTALK WITH THE CLASSICAL NUCLEAR RECEPTORS

Originally described as an “orphan” nuclear receptor [14–17] having no known ligand, the peroxisome proliferator-activated receptor-*γ* (PPAR*γ*) has since been identified as the target of the widely-used thiazolidinedione (TZD) class of antidiabetic drugs. Although the thiazolidinediones are well described as synthetic ligands of PPAR*γ*, the endogenous PPAR*γ* ligand has remained elusive. Long chain fatty acid derivatives are known to activate PPAR*γ* [18–20], but the affinity of these natural ligands for PPAR*γ* is well below the affinity of *bona fide* classical nuclear receptor ligands. However, there is now an evidence that nitric oxide derivatives of linoleic acid are potent adipogenic agonists at levels of 133 nM, well within the physiological range [21].

In vitro analyses demonstrate that various PPAR*γ* ligands (rosiglitazone, 9,10 dihydroxyoctadecenoic acid, 15-deoxy12,14-PGJ2) not only induce murine bone marrow stromal cell adipogenesis but also inhibit osteogenesis [22]. However, in vivo models suggest that not all PPAR*γ* ligands exhibit the same effects [23–25]. For example, long term treatment of mice with the thiazolidinedione troglitazone increased bone marrow adipocyte content without reducing bone mass and trabecular volume [23]. In contrast, treatment of mice with rosiglitazaone, a thiazolidinedione with higher affinity for PPAR*γ*, decreased bone mineral content, bone formation rates, and trabecular bone volume while increasing adipogenesis [24, 25].

In addition to PPAR*γ*, other nuclear hormone receptors control critical adipogenic and osteogenic steps. Among these are the estrogen and vitamin D receptors and the interplay between PPAR*γ* and these receptors has implications regarding the regulation of bone and fat formation in the bone marrow.

The effects of estrogen on bone and adipose tissue formation have long been recognized in rodent and canine ovariectomy models. In vitro studies using murine bone marrow MSCs have found that estrogen reciprocally promotes osteogenesis while inhibiting adipogenesis [26, 27]. In vitro studies using murine bone marrow MSCs have found that the soy phytoestrogen diadzein exhibits a dose dependent biphasic response: low concentrations of diadzein increase osteogenesis and decrease adipogenesis while higher doses have the opposite effect [28]. The reciprocal relationship between osteogenesis and adipogenesis is attributed to a balance between diadzein-induced activation of ER (estrogen receptor) and PPAR*γ* [28]. The importance of a balance between ER and PPAR*γ* activities is further illustrated by studies indicating that activation of PPAR*γ* with the thiazolidinedione rosiglitazone in ovariectomized rats is associated with increased bone resorption [29]. Indeed, recent studies show that a point mutation in the ligand binding domain (exon 6, C161T) of PPAR*γ* is associated with decreased levels of osteoprotegerin in postmenopausal women [30]. However, future studies are needed to determine the role of estrogen receptor and PPAR*γ* “cross-talk” in adipogenesis and osteogenesis. Estrogen can exert stimulatory effects on bone formation in the absence of the estrogen receptor alpha (ER*α*) [31]. Although estrogen-mediated changes in bone marrow adipogenesis were not determined in the absence of ER*α*, the results suggest that any reciprocal relationship between bone and fat formation may not require activation of the estrogen receptor.

Crosstalk between PPAR*γ* and vitamin D receptor (VDR) activated pathways also plays a role in the balance between bone and fat formation. The inbred SAM-P/6 (senescence accelerated mice-P/6) murine strain provides a model of accelerated senescence characterized by osteopenia and increased bone marrow fat mass [32]. Recent studies found that 1.25 (OH)_2_ vitamin D_3_ treatment inhibited adipogenesis in the SAM-P/6 mice [33]. This correlated with a 50% reduction in PPAR*γ* mRNA and protein levels as well as a decrease in Oil Red O positively stained cell numbers [33]. Additional studies indicate that 1.25 (OH)_2_ vitamin D_3_ bound VDR blocks adipogenesis by downregulating C/EBP*β* (CAAT/enhancer binding protein), a critical inducer of PPAR*γ* transcription early in adipogenesis [34]. However, ligand-free VDR appears to be necessary for adipogenesis as “knockdown” of VDR using siRNA prevents the formation of fat cells [34].

**Figure 1:**
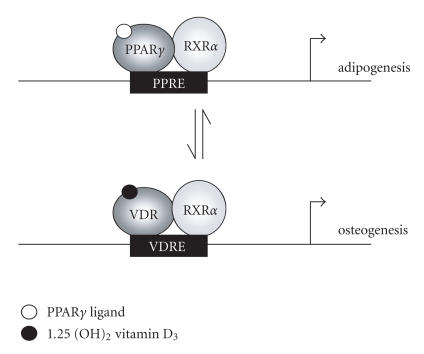
PPAR*γ* and vitamin D receptor interactions with RXR*α* may function as a switch between adipogenesis and osteogenesis.

It is tempting to speculate that the inverse relationship between adipogenic and osteogenic differentiations in the bone marrow stroma may involve competition between PPAR*γ* and other nuclear receptors such as the vitamin D receptor for their common obligate heterodimeric partner, RXR*α* (retinoid X receptor) [35] (see Figure 1). In this role, RXR*α* is well positioned to regulate the transcriptional activity of its binding partners. PPAR*γ* activity is regulated by PPAR*γ* ligands as well as the RXR*α* ligand, 9-cis-retinoic acid, even in the absence of PPAR*γ* ligand binding [36]. Indeed, adipogenesis is inhibited in the presence of 9-cis-retinoic acid in the murine TMS-14 stromal cell line [37]. Inhibition of adipogenesis is accompanied by a decrease in PPAR*γ* protein levels and suggests a decrease in PPAR*γ* transcriptional activity [37]. Conversely, VDR activity is not affected by 9-cis-retinoic acid binding to RXR*α* alone [38]. However, 1.25 (OH)_2_ D_3_-bound VDR enhances heterodimerization with RXR*α*, resulting in increased VDR activity [38]. The variable response of PPAR*γ* and VDR to RXR*α* ligand binding is consistent with the idea that RXR*α* heterodimerization may serve as a dynamic switch in the “decision” to undergo adipogenesis or osteogenesis.

## PPAR*γ* AND LXR: A CONNECTION BETWEEN LIPID METABOLISM AND BONE FORMATION

The liver X receptor subfamily of nuclear receptors, LXR*α* and LXR*β*, are pivotal in the conversion of cholesterol to bile acids. While the LXR gene was originally identified as an “orphan receptor” based on its heterodimerization with the 9-*cis* retinoic acid receptor RXR, subsequent studies identified cholesterol metabolites as endogenous LXR ligands [39]. LXR proteins are abundant in adipocytes and recent studies suggest cross-talk between PPAR*γ* and the LXRs during adipogenesis [40–43]. Although the effect of LXR agonists on adipogenesis is unclear [41, 44], several studies in murine 3T3-L1 cells link LXR to adipogenesis [41–44]. Homozygous LXR*α*/*β* ^−/−^ mice have smaller adipose tissue depots compared to their wild type littermates, suggesting that LXR regulates lipid storage [42, 43]. This effect is attributed to LXR*β* since adipose tissue is decreased in LXR*β* ^−/−^ but not LXR*α* ^−/−^ mice [43]. There is evidence that LXR activates the PPAR*γ* promoter and enhances adipogenesis in 3T3-L1 cells [44] while other studies indicate that the LXR promoter in adipocytes is regulated by PPAR*γ* [42]. These findings suggest that PPAR*γ* and the LXR proteins positively interact in the formation of adipocytes. However, LXR ligands, such as the oxysterols 20S and 22R hydroxycholesterol, inhibit adipogenesis induced by the PPAR*γ* ligand troglitazone [45]. These studies did not determine if the effects of the oxysterols in adipogenesis were LXR-mediated, leaving open the possibility that the effects are LXR-independent. It would be interesting to examine the effects of the LXR ligands on adipose tissue and PPAR*γ* activity in the LXR*α* ^−/−^ *β* ^−/−^ mouse model.

The interplay of LXR and PPAR*γ* in bone formation is relatively unexplored. While inhibiting adipogenesis, the oxysterols 20S and 22R hydroxycholesterol enhance osteogenesis [45, 46]. However, inhibition of cholesterol synthesis and presumably 20S and 22R hydroxycholesterol by the statin compounds also enhances bone formation [47], and suggests decreases in LXR ligands that are associated with osteogenesis. At present, these contradictions are difficult to reconcile and future studies examining the relationship between LXR (liganded or unliganded) and PPAR*γ* in adipogenesis and osteogenesis should provide important insights into these complex interactions.

## PPAR*γ* AND THE NUCLEAR RECEPTOR COREGULATORS: POTENTIAL ROLES IN BONE FORMATION

The transcriptional activity of the nuclear receptors is also mediated by interactions of the receptors with a large group of proteins classified as coactivators and corepressors of nuclear receptor activity. A major category of the coactivators is the p160 family of proteins that includes the cAMP response element binding protein (CBP)/p300 and steroid receptor coactivators (SRC)-1,-2,-3, which recruit histone modifiers to the chromatin structure (reviewed in [48]). A second category of coactivators includes subunits of the mediator complex such as the PPAR-binding protein (PBP)/thyroid hormone receptor-associated protein (TRAP) 220/vitamin D receptor-associated protein (DRIP) 205 [49–51]. These coactivators interact with the general transcriptional machinery to control assembly of the transcription preinitiator complex [49]. TRAP220/DRIP205, originally cloned as a coactivator of the vitamin D receptor [50], interacts directly with PPAR*γ* [51]. TRAP 220 (−/−) fibroblasts fail to undergo adipogenesis, indicating that TRAP 220 acts as a PPAR*γ*-selective coactivator [51]. An additional coactivator, peroxisome proliferator-activated receptor gamma interacting protein (PRIP), serves to link TRAP220/DRIP205 bound PPAR*γ* to the CBP/p300 coactivator [52]. PRIP (−/−) mouse fibroblasts are also refractory to PPAR*γ*-stimulated adipogenesis [53]. Although these coactivators are relatively unexplored in the regulation of osteogenesis, the essential role of PPAR*γ* in regulating the balance between fat and bone formation strongly implies a role for PPAR*γ*-coactivator interactions in osteogenesis. This possibility is supported by studies examining the effects of loss of SRC-1 [54–56]. In brown adipocytes, PPAR*γ* activity is regulated by interaction with SRC-1 and the PPAR*γ* cofactor 1(PGC-1) [57]. PPAR*γ* target genes involved in adipogenesis are decreased in SRC-1 and p/CIP (p/300 cointegrator-associated protein) knockout mice [54]. This is associated with increased metabolic rates and activity levels, indicating a role for SRC-1/PPAR*γ* interactions in energy balance [54]. Other studies using SRC-1 (−/−) mice have demonstrated that SRC-1 plays a role in bone responses to estrogen following ovariectomy, particularly in the metabolically active trabecular bone [55, 56]. Further studies will be needed to determine if SRC-1 interactions with PPAR*γ* influence responses to estrogen in metabolically active bone. However, the effects on bone formation associated with the loss of SRC-1 are expected to be complex given the general interaction of SRC-1 with nuclear receptors, including the estrogen and vitamin D receptors.

A second group of coregulators of PPAR*γ* activity are the nuclear corepressors, nuclear hormone receptor-corepressor (N-CoR) [58], and silencing mediator of retinoid and thyroid hormone receptor (SMRT) [59]. Repression of nuclear receptor activity by N-CoR/SMRT involves recruitment of histone deacetylases to the transcriptional machinery (reviewed in [60]). PPAR*γ* and VDR belong to a group of nuclear receptors that interact with N-CoR and SMRT in the absence of ligand [61, 62]. Ligand binding results in disengagement with the corepressors and recruitment of coactivators (reviewed in [60]). Studies using siRNA “knockdown” of N-CoR and SMRT in murine 3T3-L1 adipocytes show that these corepressors regulate PPAR*γ* activity during adipogenesis [63]. These results are consistent with other studies indicating that the loss of fat mass associated with calorie restriction is due to increased interaction of PPAR*γ* with N-CoR and SMRT [64]. Calorie restriction activates the histone deacetylase Sirt1, which recruits the N-CoR/SMRT corepressor to PPAR*γ* leading to inhibition of PPAR*γ* activity in adipocytes [64]. Very little is known about the effects of calorie restriction on bone formation. However, studies using resveratrol, a plant polyphenol that, like calorie restriction, activates Sirt1, may offer some insight. Recent studies in ovariectomized rats show that resveratrol treatment increases bone mineral density [65]. In addition, resveratrol increases the expression of osteocalcin and osteopontin in human bone marrow MSCs [66]. This upregulation of osteoblast markers is associated with increased responses to 1, 25 (OH)_2_ vitamin D_3_ that are accompanied by increases in expression of the vitamin D receptor [66]. These results hint at a relationship between repression of PPAR*γ* activity in adipocytes via interaction with N-CoR/SMRT and activation of vitamin D receptor responses in osteoblasts. Unraveling a potential relationship between repression of PPAR*γ* activity via interaction with N-CoR/SMRT and enhancement of bone formation may provide new therapeutic targets in treating osteoporosis in the aging population. An important area for exploration involves regulation of PPAR*γ* transcriptional activity via ubiquitin-proteasome-dependent degradation. The ubiquitin-proteasome system is responsible for the degradation of short-lived proteins in eukaryotes, including the nuclear receptors (reviewed in [67]). PPAR*γ* is targeted for degradation under basal [68] and ligand-activated conditions [69]. Recent studies show that components of the ubiquitin-proteasome system responsible for targeting substrates for degradation also function as nuclear receptor coactivators and corepressors [70–72]. Indeed, subunits of the N-CoR/SMRT complex are ubiquitin ligases that target substrates for degradation by the 26S proteasome [72]. These components, TBL1/TBLR1 (transducin *β*-like 1/transducin *β*-like 1 related protein), are required for exchange of corepressors for coactivators upon ligand binding for a number of nuclear receptors, including PPAR*γ* [72]. TBL1/TBLR1 act as adaptors for recruiting components of the ubiquitin-proteasome system to the liganded receptor [72]. In addition, deletion of TBL1 from mouse embryonic stem cells precludes the ability of these cells to undergo adipogenesis as judged by staining for neutral lipids and decreased gene expression of PPAR*γ* and PPAR*γ* targets such as adipsin [72]. Given the reciprocal relationship between adipogenesis and osteogenesis, these results suggest a role for interactions of components of the ubiquitin-proteasome system with PPAR*γ* (and other nuclear receptors) in determining the balance between bone and fat formation.

## OTHER COREGULATORS OF PPAR*γ*

Additional components of the transcriptional complex also influence PPAR*γ* activity and the differentiation of mesenchymal stem cells into either adipocytes or osteoblasts. New findings have identified a coactivator protein, known as the transcriptional coactivator with PDZ binding motif (TAZ), that is shared between Runx2 and PPAR*γ* [73, 74]. In murine cell models, the TAZ protein localized to the osteocalcin promoter in the presence of bone morphogenic protein-2 (BMP-2) and coactivated Runx2 and osteogenesis while directly suppressing PPAR*γ* and adipogenesis [73]. Although not structurally related to *β*-catenin, TAZ is proposed to be functionally similar to *β*-catenin as a regulatory switch in determining the balance between osteoblast and adipocyte development [74]. Wnt signaling stimulates osteogenesis by induction of osteogenic factors such as Runx2 [75] while suppressing adipogenesis in mesenchymal stem cells [76, 77]. Activation of the Wnt signaling pathway leads to activation of *β*-catenin, which interferes with PPAR*γ* transcriptional activity [78]. Conversely, suppression of Wnt signaling [77] and activation of PPAR*γ* [78] destabilize *β*-catenin, resulting in adipogenesis. Future studies will be needed to determine if *β*-catenin functions as a direct corepressor of PPAR*γ* activity in a manner analogous to the TAZ protein. Finally, ligand-activated PPAR*γ* itself suppresses both the expression and activity of Runx2 [79], adding another regulatory layer to the balance between bone and fat formation.

Any exploration of PPAR*γ*'s influence over bone formation must take into account the effect of oxygen tension on the development of fat and bone. It is here that the reciprocal relationship between bone and fat formation seems to disappear. The bone marrow mesenchymal stem cells (bone marrow MSC) are normally exposed to oxygen tensions lower than the atmospheric oxygen tension of 21%. In vitro studies indicate that low oxygen levels block induction of adipogenesis from human and murine MSCs [80]. Human MSCs accumulate lipid inclusions at low oxygen tensions, but the appearance of lipids is unaccompanied by expression of PPAR*γ* or the downstream PPAR*γ* target genes required for adipogenesis [81]. Adipogenesis is similarly inhibited under low oxygen conditions in human adipose-derived mesenchymal stem cells (ASC) [82]. However, reduced oxygen tension is also associated with decreased osteogenesis in the human ASCs [82, 83], suggesting parallel regulation of bone and fat development under these conditions. While hypoxic conditions (2% oxygen) do not inhibit Runx2 transcriptional activity [84], PPAR*γ* transcriptional activity is inhibited under the same conditions [85]. PPAR*γ* inhibition is mediated by HIF-1*α*, a hypoxia inducible transcription factor governing a range of cellular responses to low oxygen levels [85]. HIF-1*α* mediated repression of PPAR*γ* activity depends on an HIF-1*α* regulated transcriptional repressor, DEC1/Stra13 [85]. Interestingly, HIF-1*α*/DEC1 inhibition of PPAR*γ* under hypoxic conditions does not involve histone deacetylation, raising the possibility that the classical nuclear receptor coactivators and corepressors are not required in this process.

## CONCLUSIONS AND FUTURE QUESTIONS

These observations suggest that regulation of PPAR*γ* activity may lie at the heart of determining if bone and fat development proceed along parallel or reciprocal directions. Efforts to understand the regulation of PPAR*γ* transcriptional activity have uncovered interplay of PPAR*γ* and other nuclear hormone receptors that is intricately regulated by a range of coregulators. The coregulators extend beyond the classical coactivators and corepressors to include enzymes of the ubiquitin-proteasome system, components of the Wnt and BMP-2 signaling pathways, *β*-catenin and TAZ, and oxygen-sensing factors such as DEC1/Stra13. As research progresses in defining the role of PPAR*γ* and other nuclear hormone receptors in osteogenesis, some of the questions to be answered will include the following

1. Will new insights into MSC adipogenesis and osteogenesis be gained as the ligands for “orphan” nuclear hormone receptors are identified?
2. How do additional components of the transcriptional apparatus, such as histone acetylases and histone deacetylases, contribute to the effects of PPAR*γ* and related nuclear hormone receptors?
3. How does ubiquitin-proteasomal targeting of PPAR*γ* and related nuclear hormone receptors coordinately regulate MSC adipogenesis and osteogenesis?
4. Will these avenues of investigation have the potential to yield novel therapeutic targets or identify small molecules for osteoporosis, osteopenia, and related bone disorders?
5. Do adipokines exert either an anabolic or catabolic effect on osteogenesis?

This field of research has advanced rapidly since the discovery of PPAR*γ* over a decade ago. As new investigators are recruited to this intriguing and clinically relevant field, we anticipate that the pace of scientific progress will continue to accelerate.
